# Implementing oncology clinical trials in Nigeria: a model for capacity building

**DOI:** 10.1186/s12913-020-05561-3

**Published:** 2020-08-03

**Authors:** Atara Ntekim, Abiola Ibraheem, Adenike Adeniyi-Sofoluwe, Toyosi Adepoju, Mojisola Oluwasanu, Toyin Aniagwu, Olutosin Awolude, Williams Balogun, Olayinka Kotila, Prisca Adejumo, Chinedum Peace Babalola, Ganiyu Arinola, Oladosu Ojengbede, Christopher O. Olopade, Olufunmilayo I. Olopade

**Affiliations:** 1 Department of Radiation Oncology, College of Medicine, University of Ibadan, Ibadan, Nigeria; 2 Section of Hematology Oncology, University of Chicago, Chicago, USA; 3 Department of Radiology, College of Medicine, University of Ibadan, Ibadan, Nigeria; 4 Department of Pharmacy, University College Hospital, Ibadan, Nigeria; 5 Department of Health Promotion and Education, Faculty of Public Health, College of Medicine, University of Ibadan, Ibadan, Nigeria; 6 School of Occupational Health Nursing, University College Hospital, Ibadan, Nigeria; 7 Department of Obstetrics and Gynecology, Faculty of Clinical Sciences, College of Medicine, University of Ibadan /University College Hospital, Ibadan, Nigeria; 8 Department of Medicine, Faculty of Clinical Sciences, College of Medicine, University of Ibadan/University College Hospital, Ibadan, Nigeria; 9 Department of Pharmaceutical Chemistry, Faculty of Pharmacy, University of Ibadan, Ibadan, Nigeria; 10 Department of Nursing, Faculty of Clinical Sciences, College of Medicine, University of Ibadan, Ibadan, Nigeria; 11 Department of Chemical Pathology, Faculty of Basic Medical Sciences, College of Medicine, University of Ibadan, Ibadan, Nigeria; 12 Center for Population and Reproductive Health, College of Medicine, University of Ibadan, Ibadan, Nigeria; 13 Center for Global Health, University of Chicago, Chicago, USA

**Keywords:** Clinical trial, Oncology, Facilities, Nigeria

## Abstract

**Background:**

There is both higher mortality and morbidity from cancer in low and medium income countries (LMICs) compared with high income countries (HICs). Clinical trial activities and development of more effective and less toxic therapies have led to significant improvements in morbidity and mortality from cancer in HICs. Unfortunately, clinical trials remain low in LMICs due to poor infrastructure and paucity of experienced personnel to execute clinical trials. There is an urgent need to build local capacity for evidence-based treatment for cancer patients in LMICs.

**Methods:**

We conducted a survey at facilities in four Teaching Hospitals in South West Nigeria using a checklist of information on various aspects of clinical trial activities. The gaps identified were addressed using resources sourced in partnership with investigators at HIC institutions.

**Results:**

Deficits in infrastructure were in areas of patient care such as availability of oncology pharmacists, standard laboratories and diagnostic facilities, clinical equipment maintenance and regular calibrations, trained personnel for clinical trial activities, investigational products handling and disposals and lack of standard operating procedures for clinical activities. There were two GCP trained personnel, two study coordinators and one research pharmacist across the four sites. Interventions were instituted to address the observed deficits in all four sites which are now well positioned to undertake clinical trials in oncology. Training on all aspects of clinical trial was also provided.

**Conclusions:**

Partnerships with institutions in HICs can successfully identify, address, and improve deficits in infrastructure for clinical trial in LMICs. The HICs should lead in providing funds, mentorship, and training for LMIC institutions to improve and expand clinical trials in LMIC countries.

## Background

Cancer, once considered the disease of high income countries (HIC), has slowly become endemic in low income countries (LMIC). Although Westernized lifestyle may be contributing to this surge in cancer incidence, the accompanying higher mortality rates in these vulnerable populations is alarming [1]. Compared to HICs, patients in LMICs present more often with locally-advanced stage or metastatic cancer due to patients’ and health care providers’ lack of knowledge and understanding of the disease, as well as the paucity of data on the biology of cancer in patients of African descent [2, 3]. Providing cancer care in this environment has many challenges, such as lack of health care infrastructure, clinical expertise, research infrastructure, human resources and non-implementation of health policies [4, 5]. To bridge the cancer geographical divide and improve quality of cancer care at affordable costs, diagnostic and therapeutic approaches are needed. Now is the time to accelerate progress in combating the looming epidemic of breast cancer in these LMICs that are least prepared to bear the burden of the disease [6, 7] .

**Fig. 1:**
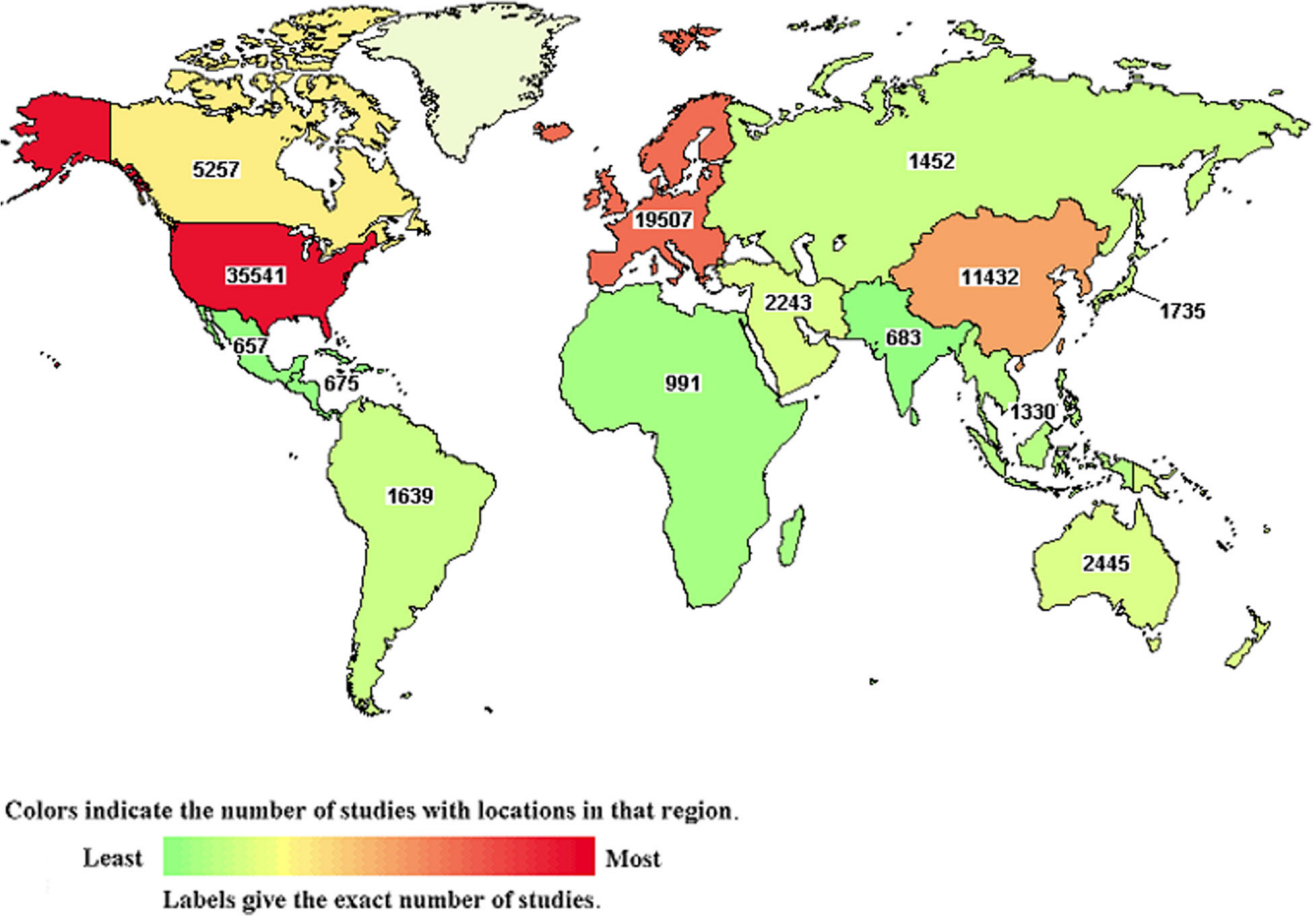
Distribution of study locations of oncology clinical trials registered with ClinicalTrials.gov, showing comparatively low levels of clinical trial studies in LMICs compared with HICs. Africa has 991 out of world total of 44,772,546 studies (< 2%) (Source: U.S. National Library of Medicine. https://clinicaltrials.gov/ct2/results/map?cond=Oncology&map= (Accessed January 29, 2020).

Clinical research drives the field of oncology. Clinical trials have traditionally been carried out in relatively resource-rich locations, such as North America, even though the majority of cancer patients live in low resource settings [8]. In recent years, a shift in location of Biopharma industry-sponsored clinical trials to regions such as Eastern European, Latin American, Asian countries and South Africa has occurred largely due to national policies in these emerging markets [9–11] An expanded globalization of innovative biomarker-informed oncology clinical trials to include countries in Africa is long overdue. However, despite the significant link to African Americans in the US, there are few clinical trials conducted in Sub-Saharan Africa [12–14] (Fig. 1).

While lack of infrastructure, resources, medical expertise and incentive to the pharmaceutical industry have been cited as barriers [15], other industries including digital media and financial services, appear to be thriving on the Continent. Breakthroughs in the treatment of infectious diseases, such as HIV/AIDS in Nigeria, have greatly impacted and improved care for people living with HIV. Nigeria successfully responded to the Ebola Epidemic using a vast network of public health experts, demonstrating that clinical trials in West Africa, particularly in Nigeria, is feasible [16] and should be pursued as a global strategy to address the rising epidemic of cancer in Africa and increase health care equity.

Opportunities to unravel the root causes of disparities in cancer outcomes, based on geography and/or genetic ancestry, exist in involving Sub-Saharan Africa in conducting biomarker driven oncology clinical trials. Africa is the cradle of humanity and learning about the diversity of genomes and geography in which cancer occurs could create breakthroughs for drug development. This can also serve as a bidirectional transfer of knowledge as this could lead to implementing interventions that include Blacks made up of African American patients (as well as Blacks in other diaspora settings) in clinical trials conducted in the United States and other countries.

With the goal of leapfrogging towards quality cancer care, Nigeria partnered with the University of Chicago to initiate an oncology clinical trial program in collaboration with four institutions in Southwestern Nigeria, including the University of Ibadan, the University of Lagos, Lagos State University and Obafemi Awolowo University, Ile-Ife, Nigeria.

Prior to these partnerships, a study was conducted to assess the capacity and infrastructure available for conducting biomarker-driven clinical trials in these four institutions. The assessment was done with a checklist developed using Site Assessment Questionnaire (Pre-visit) Tool developed by the National Institute of Dental and Craniofacial Research (NIDCR), National Institutes of Health USA (https://www.nidcr.nih.gov › sites › default › files › site-assessment-questio..) and the Site Assessment/Feasibility Questionnaire of Global Health Network (https://globalhealthtrials.tghn.org › site_media › media › articles › Site_Ass.).

The aim of the study was to identify areas where intervening might improve the ability to conduct innovative biomarker-driven clinical trials locally, and to build capacity that could lead to improved cancer care in Nigeria. This study was carried out with the following objectives: 1) To identify needed facilities to be put in place that will fully support the conduct of oncology clinical trials in four centers in Nigeria; 2) To document available trained manpower for oncology clinical trials in selected sites in Nigeria and 3) To identify training needs of oncology research teams in Nigeria. The outcome measures were the facilities upgraded, the number of personnel trained and the number of sites that were capable of anchoring oncology clinical trials in Nigeria.

## Methods

### Engagement of stake holders

We first engaged with stakeholders, key opinion leaders, policy makers such as departmental heads, Chief Medical Directors (CMDs) of four hospitals, Provosts of two Colleges of Medicine, Commissioners for health, Governors of two states who were associated with selected hospitals and the Federal Ministry of Health. The relationship fostered with these Key Opinion Leaders and policy makers were instrumental to clinical trial capacity building. They were interested in developing the capacity of Nigerian researchers to conduct interventional cancer research in addition to other ongoing research activities such as observational studies. The Center for Global Health, University of Chicago, has been collaborating with four institutions in Nigeria in biomedical research. It was thought needful to further develop research capacity in clinical trial as part of the strategy to control the rising burden of cancer diseases in sub-Saharan Africa (SSA). In addition to NIH research grant funding, the center reached out to other organizations namely Breast Cancer Research Foundation (BCRF) USA, Novartis Institute for Biomedical Research (NIBR) USA and F. Hoffmann-La Roche & Co Switzerland. These organizations supported the concept in order to assist in improving clinical trial infrastructure that will enable clinical trials led by indigenous researchers to be conducted in SSA. This is based on the premise that the best way to increase the conduct of clinical trials in SSA is to train local investigators and improve facilities whereby they will be involve in designing and conducting studies that will be relevant to the population.

### Site survey and assessment

Four oncology centers in South West Nigeria were selected for this survey. The selection was based on previous research collaborations on biomedical research between the four Nigerian sites and the University of Chicago. A site questionnaire was sent to each site’s principal investigator to obtain basic information about oncology clinical trial facilities available. The questionnaire elements included basic site information such as name, address and location, site resources, qualification of principal investigator and co-investigators, IRB/other review committees, other clinical trial team members and previous involvement in clinical trials. Following this, requests for site assessment visits appointments were made to the principal investigators of each site for site assessment visits on selected dates and time and approvals were received. Visits were made to each of the sites to confirm information from the site questionnaire and verify site facilities. In addition, needs assessment were carried out at the four collaborating teaching hospitals in Nigeria to identify and assess resources such as infrastructure, expertise, care operative procedures available for the conduct of biomarker-driven oncology clinical trials. The team members conducting the assessment were staff from the University of Chicago Center for Global Health, the clinical trial team of a pharmaceutical company, and investigators from member institutions namely the University of Ibadan, the University of Lagos, Lagos State University and Obafemi Awolowo University. A checklist of clinical trial activities was prepared for use in the assessment exercise. The list included availability of diagnostic services such as blood tests and radiological investigations, pharmacokinetic studies, clinical investigators, study coordinators, oncology nurses, availability of chemotherapy preparation and administration facilities, oncology pharmacy services and capabilities in handling investigational medicinal products (IMPs). Other items on the checklist included availability of standard operating procedures (SOPs) for various procedures, quality of life assessment services, handling of bio-specimens, filing and storage of patients’ data, availability of study monitors and existence of Institutional Review Boards.

### Site visits and training on GCP

The visits were made in February 2017 and lasted 7 days with 1 day spent at each center. At each center, visits were made to the oncology clinics, oncology pharmacies, chemotherapy preparation rooms, chemotherapy administration facilities, pharmacokinetic specimen collection rooms and analysis facilities, clinical laboratories, radiology units, Echocardiography and ECG suits, surgical theatres, admission wards, radiotherapy facilities, pathology/molecular pathology facilities and specimen storage facilities. At each facility, the functionality of the equipment was ascertained as well as the calibration and maintenance status. Information on the operations of institutional review boards (IRBs) was also obtained. At the end of each site assessment visit, there were interactions with local team members as well as the management of each center. The interactions were to enable the inspection team to assess the experience and training of team members, identify needed expertise in the teams and identify training needs. Identified gaps were enumerated at each center. There were discussions and plans on how to improve the facilities. Joint training sessions on essentials of clinical trials including GCP were conducted during the remaining days. Following the first visit, several deficiencies were noted, and the upgrade of facilities were commenced with online training of team members on clinical trials carried out by facilitators from the University of Chicago. There was a repeat site visit to assess the progress of work in May 2018 by the collaborators followed by interactions with the local team members and management of each center, as well as onsite training. Further efforts were made to bring facilities to acceptable standards.

## Results

During the site assessment visits, deficiencies were noted and plans were made to address them to ensure the sites were prepared to participate in clinical trials. After completion of the initial site assessment visit of all four local institutions, the following Infrastructural deficits were identified (Table 1).

**Table 1:** State of facilities for oncology clinical trials before and after intervention at four centres in Nigeria

Pre-intervention facilities					Post- intervention facilities			
	***UCH***	***LASUTH***	***OAUTH***	***LUTH***	***UCH***	***LASUTH***	***OAUTH***	***LUTH***
*Clinical evaluation*								
Clinic instruments	No calibrations	No calibrations	No calibrations	No calibrations	Yearly calibrations	Yearly calibrations	Yearly calibrations	Yearly calibrations
Pathology Labs	Available	Available	Available (No IHC)	Available (No IHC)	IHC available	IHC available	IHC available	IHC available
Minus 80 Freezer	Available	Available	Available	Available	Available	Available	Available	Available
Imaging (CT)	Down	Down	Available	Available	Available	Available	Available	Available
*Spaces*								
CTH rooms	Nil	Available	Available	Nil	Available	Available	Available	Available
CTH preparation	Nil	Nil	Nil	Nil	Available	Available	Available	Available
IMP storage	Available	Nil	Nil	Nil	Available	Available	Available	Available
Data Storage	Available	Available	Nil	Nil	Available	Available	Available	Available
Emergency facilities	Poorly maintained	Poorly maintained	Poorly maintained	Poorly maintained	Good	Good	Good	Good
*Personnel*								
Oncology nurses	4	3	2	4	8	6	6	8
Onco-pharmacists	1	Nil	Nil	Nil	4	2	2	2
Study Coordinators	2	Nil	Nil	Nil	3	2	2	2
GCP trained staff	3	Nil	Nil	Nil	14	10	10	10
Data Managers	2	2	1	Nil	3	2	2	2
Clinical Trial Manager	Nil	Nil	Nil	Nil	2 (for the network)	–	–	–

*CTH* chemotherapy, *IHC* immuno-histochemistry, *IMP* investigational medical products, *UCH* University College Hospital, *LASUTH* Lagos State University Teaching Hospital, *OAUTH* Obafemi Awolowo University Teaching Hospital, *LUTH* Lagos University Teaching Hospital

### Clinical evaluation, laboratory, and imaging studies

All four centers had basic functioning apparatus to measure vital signs such as thermometers for body temperature, blood pressure (BP) apparatus, weighing scales and height measurement but there were no regular yearly calibrations and maintenance of these items at any of the sites.

Two out of the four centers had standard blood investigation laboratories with Standard Operative Procedures, regular quality assurance checks, equipment maintenance logs and participation in international certification programs. The four centers had well-established pathology departments and services but there were issues of equipment malfunction or lack of reagents for certain investigations such as immunohistochemistry in some of the pathology laboratories. Facilities for pharmacokinetic studies were available at all four centers with qualified personnel. However, there were different inventories of analytic equipment such as spectrophotometer and high-performance liquid chromatography (HPLC) equipment across the centers. Ultrasound equipment and echocardiogram machines were available at all four centers, but none had evidence of yearly maintenance checks. Functioning CT scan machines were available at two of the four centers. The other two centers had broken CT scan equipment, although there were plans to repair them.

### Oncology nursing staff and chemotherapy facilities

All four centers had oncology nurses, although few in number; and none had prior experience with clinical trials. There were dedicated chemotherapy administration rooms in only two centers. All the centers had emergency and resuscitation facilities but with incomplete inventories and lack of regular stock checks of items. Quality of life assessment services were available in one center, though they were not routinely offered to regular cancer patients. Chemotherapy preparatory facilities with biosafety hoods were lacking in all centers visited. There were no infusion pumps for chemotherapy delivery in any of the centers.

### Pharmacy personnel, drug procurement and management of drug inventories

Only one center had an oncology pharmacist with experience in oncology drug preparation, drug accountability, drug storage, inventory and maintenance of logs. Standard investigational medical products (IMPs) storage facilities were available in one center; however there was no alarm system notifying staff about change in temperature in cold chain therapies. This was however, manually done by performing regular checks even during non-office hours. There was no special procedure for disposal of remnants of oncology drugs at any of the centers. These were handled in the same manner as other hospital wastes.

### Skills of local investigators and clinical trial staff

Skills were assessed based on previous Good Clinical Practice (GCP) training and knowledge on safety reporting standards. Among the four centers, one investigator and two study coordinators had prior training in GCP. No other study personnel had GCP training but investigators from all the centers had training in the ethical conduct of research, including the handling of human subjects in clinical research. Administrative staff for trial administrative functions were lacking in all the centers. None of the centers had an experienced Trial Monitor capable of conducting internal monitoring of trial activities.

### Data management processes and data monitoring

Two centers had facilities for clinical research documentation and data storage. Three centers had data management staff, but none were trained to maintain trial records and other important clinical trial and IRB-related documents. Robust online data transmission facilities were lacking in two centers.

### Storage of biospecimens

All the centers had facilities for the storage of biospecimens collected from clinical trials although some had only limited space. Standard SOPs were lacking in all the biospecimen storage facilities.

### Institutional review boards

The four centers had well-constituted, active ethical review boards. They had good experience in monitoring non-oncology clinical trials. Given the dearth of oncology clinical trials in Nigeria, these boards’ expertise specific to this domain could not be assessed.

### Standard operating procedures (SOPs)

SOPs were available only in the two standard clinical laboratories identified. The pathology laboratories of the four centers had SOPs for procedures, such as immunohistochemistry studies, sample storage and processing. Other service areas such as radiology, pharmacy, clinical services sections, including chemotherapy administration services and outpatient clinics, as well as the informed consent process, had no SOPs.

### Interventions

Following the identification of the infrastructural deficiencies, the University of Chicago led in providing and sourcing grants and in identifying partners that could help address the deficiencies. These were sourced from NIH through D43 and K43 grant awards, philanthropic funding from the Breast Cancer Research Foundation and collaborations with pharmaceutical industries such as Novartis and Roche Pharmaceutical. Using these resources, improvements in institutional capabilities and facilities were carried out in the following areas:

### Training of personnel

Two investigators were trained in study protocol development and trial management and five pathologists were trained in accurate and standard tissue diagnosis, reporting, and associated laboratory procedures. Those trained also included two radiologists, three study coordinators, one counselor and an oncology pharmacist. These personnel were trained in the USA (Table 2). They returned to Nigeria to train others in their respective fields. These trained personnel then trained at least three personnel in their respective fields of specialization.

**Table 2:** Types of training in the USA received by members of the clinical trial team from four institutions in Nigeria

Personnel	Number trained	Training Institution	Duration (months)	Training focus
*Clinical investigators*				
Clinical Oncologist	2	(i) NIBR, (ii) U Chicago	3 each	Protocol development, Clinical trial management
Medical Oncologist	1	University of Chicago	36	
Pathologists	5	University of Chicago	3	Accurate and standard tissue diagnosis, reporting and associated laboratory procedures, GCP
Laboratory Scientists	4	2- University of Chicago 2- NIBR	3 3	IHC techniques SOP Development, GCP
Radiologists	2	University of Chicago	3	Breast imaging and USS guided biopsy, GCP
Study Coordinators	3	University of Chicago	3	GCP, Participants’ recruitment, Adverse events monitoring
Oncology Pharmacist	1	University of Chicago	3	Drug accountability, storage, preparation of chemotherapy agents, GCP
Genetic Counsellor	2	University of Chicago	6	Counselling techniques, GCP
Pharmaceutical chemists	2	University of Chicago	3	Pharmacokinetics/pharmacogenomics, GCP

*NIBR* Novartis Institute for Biomedical Research, Cambridge Massachusetts, *GCP* good clinical practice, *IHC* immunohistochemistry, *SOP* standard operating procedure, *USS* ultrasound

In addition, two local training workshops were conducted in Nigeria facilitated by staff from the University of Chicago and Roche Pharmaceutical Company. These training sessions were on Good Clinical Practice, ethics in clinical research and Standard Operative Procedure development. Sessions were well attended by members of the clinical trial teams from the four institutions. The training topics included steps and processes involved in conducting clinical trials, such as informed-consent, handling of IMPs, participants’ screening, recruitment and follow up. Other in-country training sessions were conducted for eight study monitors; two of these trainees were designated to serve as internal monitors while six functioned as external monitors for the clinical trial activities of the network.

### Upgrade of facilities

In all four institutions, oncology drug storage, drug preparation room (clean room), biosafety cabinets and therapy administration facilities were established (Table 1). All four institutions supported these efforts by internally-sourcing funds to renovate and improve their respective cancer treatment units. There was also commitment to regular servicing and calibration of equipment used for patient care and clinical trial activities. The support and commitment in all institutions were facilitated by Key Opinion Leaders and Policy makers whose interest was to upgrade the ability of their respective institutions to conduct interventional cancer clinical trials as well as other kinds of trials that can ultimately improve the care of patients or has the tendency to expose patients to innovative interventions. The clinical trial capacity of the country particularly in these four indexed institutions is rising therefore there are local aspirations to be a part of the globalization of clinical trials. Pharmaceutical companies who have been excluding SSA populations from clinical trials citing poor infrastructure as the reason, can now start thinking of including African sites in their drug development programs. In comparison with conducting trials in the Western world, conducting trials in LMIC particularly in Nigeria would be cost effective at the same time having the potential of improving the healthcare system.

## Discussion

### Clinical trial readiness

The partnership between the University of Chicago and four institutions in Nigeria were deemed successful, with these types of partnerships between academic institutions in high income countries with institutions in low income countries as key to improving clinical trial infrastructure and participation among LMICs [17]. Specifically, outcomes of this partnership were: (i) locally trained providers with the ability to conduct clinical trials, (ii) upgraded infrastructures, and (iii) skills acquisition in clinical trial protocol writing and (iv) the development of the clinical trial unit (CTU) which comprises of local healthcare providers such as physicians, pharmacists, nurses and administrative support personnel. All the four institutions have Oncology Clinical Trial Units consisting of highly skilled providers with the capability of collaborating with other institutions, countries, and teams to conduct biomarker driven cancer clinical trials locally. Three protocols on investigator-initiated biomarker driven oncology trials written by local investigators have now been completed. The first study the ARETTA Study (Assessing response to neoadjuvant Taxotere and Trastuzumab in Nigerian women with HER2-positive breast cancer **(**ClinicalTrials.gov Identifier: NCT03879577) is now being conducted at the four centers in Nigeria under the sponsorship and close supervision of the University of Chicago USA. Without these interventions, this study would not have been possible. Following the successful conduct of this first clinical trial, additional lessons learnt will help to further consolidate clinical trial activities in Nigeria.

### Capacity building

In addition, there was an improvement in clinical practice and the care operative procedure for all cancer patients reported in all institutions in the following ways.

(i) The clinical laboratory values have been standardized in all institutions with regular quality assurance checks being done on all equipment. All the pathology laboratories now have SOPs for all procedures with well trained personnel in conducting immuno-histochemistry studies, with a regular supply of reagents to ensure that these tests are routinely carried out especially on all breast cancer specimens.
(ii) Per protocol, care of trial participants has been improved, which is crucial for the validity of clinical trial results. Our clinical care providers, particularly nurses, know how to look for, record, and report patients’ signs and symptoms. Ability to handle adverse events is closely associated with good performance of clinical trials.
(iii) These outcomes support Weigmann’s (2015) findings that clinical trials have the added benefit of building research and health care capacity and can improve local facilities and improve the economy as well as teamwork and collaboration [18].
(iv) All the participating centers have skills and facilities for the preparation, administration and disposal of chemotherapy agents. This will lead to adequate protection of patients, staff and the general public.
(v) Record keeping facilities and personnel skills have been improved and all the centers now have adequate record keeping procedures.
(vi) There are SOPs for all relevant procedures at all centers. This ensures uniformity in carrying out procedures that will result in uniform output.
(vii) All the centers now have well trained clinical trial team in place.
(viii) The preparation of institutions for clinical trials comes with improvement in cancer clinical practice and this has been observed as more interdisciplinary team collaborations, availability of acquired infrastructures for all patients, improved knowledge of oncology nurses and pharmacists translating to better care and more accessible pathology services. Whilst this paper hones on the preparedness of institutions for oncology clinical trials, as distinct from clinical practice which they have been carrying out, the future direction of our group is to quantify the direct and indirect benefits of clinical trial to the local practice

Members are well versed in ethical issues in conducting human research as well as good clinical practice. Prior to this intervention, there was no trained study monitors and clinical trial manager. Eight study monitors have so far been trained as well as two clinical trial managers for the network. The University of Chicago also granted access to her web services for the four centers to use for regular web meetings. Through this, there is continuous collaboration between team members in the four institutions and within the institutions as well as the University of Chicago thereby improving team building. It has been reported that the collaboration with HIC institutions and investigators have a high probability of bringing expertise, funding, and resources to SSA [17]. Furthermore research centers in LMICs benefit from partnering with externally sponsored clinical trials in terms of building capacity and investment [19]. Activities in this study have shown that collaboration between HIC and LMIC institutions is a pathway to the development of oncology clinical trials cooperative group in West Africa. Although this model was tested with clinical trials involving medications, the approach can be adapted in non-drug related clinical trials by approaching relevant HIC partners for support. It is also important to note that identifying HIC agencies with shared interest in partnering with LMIC institutions towards developing clinical trial infrastructure as a means of improving cancer care in LMICs is key to success.

## Conclusion

Gaps responsible for the lack of involvement of the four centers in oncology clinical trials included poor patient care skills, infrastructural deficits, and lack of funding. Collaborative interventions with institutions from HICs corrected these on-going issues. The four centers now have well-trained clinical investigators who, although dealing with low-standard facilities, can conduct clinical trials to international standard using available facilities. Our approach can therefore serve as a model for improving facilities for oncology clinical trials in Sub-Sahara Africa. In addition, it can serve as a blueprint to centers in HICs who are passionate about improving participation in oncology clinical trials in LIMCs such as Sub-Sahara Africa. Centers in LMICs interested in positioning themselves for inclusion in clinical trials should be ready to undergo needed transformation as described in this report.

## Data Availability

The datasets used and/or analysed during the current study available from the corresponding author on reasonable request.

## Abbreviations

BCRF: Breast Cancer Research Foundation
BP: Blood pressure
CMDs: Chief Medical Directors
CTH: Chemotherapy
CTU: Clinical trial unit
GCP: Good Clinical Practice
IH: Immuno-histochemistry
IMPs: Investigational Medicinal Products
IRB: Institutional Review Boards
HICs: High Income Countries
HPLC: High-performance liquid chromatography
LASUTH: Lagos State University Teaching Hospital
LUTH: Lagos University Teaching Hospital
LMC: Low and Medium income Countries
NIBR: Novartis Institute for Biomedical Research
NIDCR: National Institute of Dental and Craniofacial Research
NIH: National Institutes of Health
OAUTH: Obafemi Awolowo University Teaching Hospital
SOP: Standard Operating Procedures
SSA: Sub-Sahara Africa
UCH: University College Hospital
USS: Ultrasonography
